# What have we learned?

**DOI:** 10.7554/eLife.75830

**Published:** 2021-12-07

**Authors:** Peter Rodgers, Andy Collings

**Affiliations:** 1 eLife Cambridge United Kingdom

**Keywords:** Reproducibility Project: Cancer Biology, replication, reproducibility, open science, meta-analysis, reporting standards

## Abstract

As the final outputs of the Reproducibility Project: Cancer Biology are published, it is clear that preclinical research in cancer biology is not as reproducible as it should be.

Back in 2014, when the first articles from the Reproducibility Project: Cancer Biology (RPCB) were published in eLife, there were widespread concerns about what seemed to be low levels of replicability and reproducibility in some areas of research. Researchers at two drug companies – Bayer and Amgen – had reported that they had not been able to replicate many published findings in cancer biology and other areas of preclinical research (Prinz et al., 2011; Begley and Ellis, 2012). Since then large-scale studies of replicability and reproducibility in psychology, economics and other areas of research (Open Science Collaboration, 2015; Camerer et al., 2016), reports from learned societies (Academy of Medical Sciences, 2015; NAS, 2019), surveys of researchers (Baker, 2016; Boulbes et al., 2018), and popular books (Harris, 2017; Ritchie, 2020) have ensured that concerns about the 'reproducibility crisis' have maintained a high profile ever since.

The RPCB had two main aims: to provide evidence about replicability in preclinical cancer research, and to identify the factors that influence replicability more generally. Now, seven years later, the final three articles from the project have just been published, and they confirm that there is still considerable scope for improving the reproducibility of preclinical research in cancer biology (Errington et al., 2021a; Errington et al., 2021b; Errington et al., 2021c).

The RPCB was a collaboration between the Center for Open Science and Science Exchange, and the project was funded by a grant from a private foundation (now called Arnold Ventures). To achieve its aims the project team planned to repeat selected experiments from 53 high-profile papers in the field of cancer biology that had been published in the period 2010–2012. eLife agreed to be the publishing partner for the project, and to use what was then a new approach to peer review to assess the outputs of the project.

Under this approach, for each paper selected, the project team would prepare a Registered Report that described in detail how the experiments would be carried out and how the data would be analyzed. Each Registered Report would be peer reviewed, and experiments could not begin until it had been accepted for publication. The results of the experiments would then be written up as a Replication Study, which would be peer reviewed to ensure that the experiments and data analysis had been performed in accordance with the Registered Report. Where possible one of the authors of the original paper would be involved in the peer review of both the Registered Report and the Replication Study.

A total of 193 experiments from 53 papers were selected for replication, and the project team set about preparing Registered Reports for each paper. However, as recounted in detail in 'Challenges for assessing replicability in preclinical cancer biology' (Errington et al., 2021a), the team encountered problems almost immediately. For example, many of the original papers failed to report key descriptive and inferential statistics, and despite contacting the original authors the project team was unable to obtain these data for 68% of the experiments. Similarly, none of the 193 experiments were described in sufficient detail for the project team to design protocols to repeat them. And although the original authors were often helpful when asked for such details, they were 'not at all helpful' (or did not respond to the project team) for 32% of the experiments. These problems meant that the early stages of the project took longer than expected and went over budget: the end result was that it was only possible to publish 29 Registered Reports.

Once experimental work started, two-thirds of the protocols needed to be modified to allow the experiments to be completed. Again this stage of the project took longer and cost more than expected, and in the end the project team was only able to repeat 50 experiments from 23 papers: the results of these experiments are reported in 17 Replication Studies and an aggregate paper (Errington et al., 2021c). The clear message to emerge here is that the reporting of both methods and results needs to be improved.

So how replicable were the 50 experiments that the team managed to repeat? As explained in a meta-analysis that combines the data from all the replications (Errington et al., 2021b), there are a number of different answers to this question. One reason for this is that many of the experiments involved measuring more than one effect (such as measuring the influence of an intervention on both the tumor burden and overall survival). Indeed, the 50 experiments involved a total of 158 effects. Moreover, these effects could be positive effects or null effects. Furthermore, some of the original papers reported effects in terms of numerical values, whereas others relied on images.

The team used seven criteria to assess replicability, although some were not suitable for assessing all effects (e.g., some only worked for positive effects, or when numerical values were available). One criterion compared effect sizes for positive effects: this revealed the median effect size in the replications was 85% smaller than in the original experiments; moreover, the effect size in the replication was smaller than the original in 92% of cases. The other criteria were binary – the replication was either a success or a failure – and five of these could be used for both positive and null effects when effect sizes were reported as numerical values. For positive effects, 40% of replications succeeded according to three or more of these criteria, and this figure increased to 80% for null effects.

In a separate article, Patrick Kane and Jonathan Kimmelman (who were not part of the RPCB) take a step back and discuss some of the scientific, ethical and policy implications of the project (Kane and Kimmelman, 2021). They liken basic and preclinical research in cancer biology to a 'diagnostic machine' that is used to decide which clinical hypotheses should be progressed (including which should go forward to clinical trials). While the results of the RPCB may be 'concerning', Kane and Kimmelman argue that further work is needed to better understand the performance of the diagnostic machine.

And further work is being done on many fronts. National projects to explore various aspects of reproducibility are under way in several countries, including Brazil (Amaral et al., 2019; Amaral and Neves, 2021), Germany (BMBF, 2018) and the Netherlands (NWO, 2020). National reproducibility networks have also been set up in Germany and the UK.

The aim of the RPCB was not to find papers that were flawed or faulty, and a failure of the team to replicate an experiment does not mean that the original was wrong (and, likewise, a successful replication does not guarantee that the original was correct – both the original and the replication may be wrong). However, the results of the project should give the biomedical research enterprise pause for thought. Journals have encouraged more complete reporting of methods and results in recent years, but there is still scope for improvement, especially when it comes to making data and code openly available. Many studies would benefit from greater input from experts in statistics, ideally before data are collected, and preregistration should help to reduce bias and increase rigor in certain types of studies. Increased preprinting will also help for most papers by increasing both readership and scrutiny, and by making new results available sooner. Lastly, a greater emphasis on science that is rigorous, as opposed to eye-catching, from researchers, institutions, funders and journals would benefit everyone.

## Note

All *eLife* content related to the Reproducibility Project: Cancer Biology is available at: https://elifesciences.org/collections/9b1e83d1/reproducibility-project-cancer-biology.

All underlying data, code, and digital materials for the project is available at: https://osf.io/collections/rpcb/.
